# Immune Investment Is Explained by Sexual Selection and Pace-of-Life, but Not Longevity in Parrots (Psittaciformes)

**DOI:** 10.1371/journal.pone.0053066

**Published:** 2012-12-27

**Authors:** Darryl B. Edwards

**Affiliations:** Department of Biology, Laurentian University, Sudbury, Ontario, Canada; Arizona State University, United States of America

## Abstract

Investment in current reproduction should come at the expense of traits promoting future reproduction, such as immunity and longevity. To date, comparative studies of pace-of-life traits have provided some support for this, with slower paced species having greater immune function. Another means of investment in current reproduction is through secondary sexual characters (SSC). Investment in SSC's is considered costly, both in terms of immunity and longevity, with greater costs being borne by species with more elaborate traits. Yet within species, females prefer more ornate males and those males are typically immunologically superior. Because of this, predictions about the relationship between immunity and SSC's across species are not clear. If traits are costly, brighter species should have reduced immune function, but the opposite is true if SSC's arise from selection for more immunocompetent individuals. My approach was to investigate immune investment in relation to SSC's, pace-of-life and longevity while considering potentially confounding ecological factors. To do so I assessed leukocyte counts from in a novel group, the Psittaciformes. Investment in SSC's best explained investment in immunity: species with brighter plumage had higher leukocyte counts and those with a greater degree of sexual dichromatism had fewer. Ecological variables and pace-of-life models tended to be poor predictors of immune investment. However, shorter incubation periods were associated with lower leukocyte counts supporting the notion that species with a fast pace-of-life invest less in immunity. These results suggest that investment in reproduction in terms of fast pace-of-life and sexual dichromatism results in reduced immunity; however, investment in plumage colour per se does not impose a cost on immunity across species.

## Introduction

The immune system has evolved in response to energetic trade-offs with other life history traits and with the risk of infection. The balance between maintaining optimal immune defence and aspects of life history, such as reproduction, has generated considerable research in the past couple of decades (e.g., [1], [2].

The fundamental prediction of life history theory is that individuals trade-off among activities that compete for resources, such as reproduction and survival [3], [4]. Since immune function is an important aspect of self-maintenance that is energetically costly [5], [6], investment in immunity should be traded against investment in reproduction [7], [8]. For many species, emphasis on reproduction produces exaggerated secondary sexual characters (SSC) that compete for resources that would otherwise be used for self-maintenance. For example, adult mortality appears to be related to costs associated with bearing SSC's, such as large body size in mammals [9], [10] or bright plumage in birds [11], [12]. In the case of birds, costs associated with bearing bright plumage are considered a fundamental aspect of signalling theory [13], and the notion that elaborate signal traits impose costs directly on the immune system is well established [8], [14].

If investment in SSC's is costly, we might expect these traits to be negatively related to immune investment and longevity across species. Yet within avian species, males with more elaborate plumage are preferred by females [15] and are often immunologically superior to, and have fewer parasites than duller individuals (e.g., [16]). Across species, those with more parasites tend to have brighter plumage [17], possibly resulting from stronger selection for more immuno-competent individuals in species experiencing higher infection rates (although the reverse argument can also be employed: costly plumage traits could result in reduced immune investment across species resulting in high rates of parasitism). As a result, brighter species could have greater immune investment than duller ones, and consequently greater longevity, which is contrary to the notion of costly investment in SSC's.

The main goal of this study is to address a paradox of sorts: do species investing in elaborate plumage traits face costs for doing so and hence have reduced immunity, or does plumage exaggeration signal greater immune ability against increased infection threats? I measured sexual selection in terms of overall plumage exaggeration, as well as the degree of sexual size dimorphism and dichromatism. I predict that species with more elaborate plumage will have greater immune investment, in keeping with the idea that greater intra-sexual selection for good quality individuals results in more elaborate plumage at the species level. Alternatively, I predict that more pronounced sexual dimorphisms in body size and plumage result in reduced immune investment as a consequence of conflicting sexual strategies. For example, in the studies cited above, costs reported for SSC's were measured as sexual size dimorphism [9], [10] and sexual dichromatism [11], rather than overall expression (but see [12]). Sexual dimorphisms in appearance are likely to evidence sexual conflict for other traits resulting in compromises to investment in self-maintenance at the species level [18].

A secondary goal of this paper is to place sexual selection and longevity in the context of other life history traits more typically studied in the recent comparative eco-immunology literature. Longevity is rarely used in these studies, and instead immune investment is often investigated in the context of traits associated with pace-of-life. Pace-of-life is directly related to longevity: a slow pace-of-life is typified by species with long developmental periods and late maturation, low reproductive potential and long life span [19]. On the fast end of the life history continuum, species are expected to invest more in reproduction, such as increased clutch size and possibly SSC's [20], and less in immunity. However, to date the results from pace-of-life studies have been somewhat disparate with slower pace-of-life showing both superior and inferior immune function depending on the measure of immunity used [21], [22], [23], [24].

The relationship between plumage colour and parasites demonstrated by Hamilton and Zuk [17] was initially criticized because it lacked potentially confounding ecological variables that could influence either plumage colour or infection risk [25]. Because of this I included four ecological variables – group size, habitat, diet, region – that could help explain patterns in plumage colour, infection risk and longevity.

Although the interplay between infections and immunity is complex, the immune system is expected to evolve in concert with the risk of infection. Transmission rates of infections increase with group size [26], and as a consequence so should investment in immunity. In support of this, immune investment is higher in species forming larger social groups (birds: [27], mammals: [28], [29]) and with increased mating promiscuity (mammals: [28], [29]). However, infection rates could also be indirectly related to plumage colour if signal intensity increases with social group size as a means of communication among individuals in large flocks.

For habitat, diet and region specific expectations are not as clear: *Habitat*) The perception of colour depends upon the nature of the light environment, and the light environment differs with light penetration in different habitats [30]. In order to maximize signal efficiency, selection on plumage colour differs with habitat use [31], where habitat use is defined in the context of light penetration. *Diet*) Dietary antioxidants, such as from fruit, can play an important role in bolstering immunity [32]. Diet can explain patterns in longevity as well, with the degree of frugivory accounting for both shorter [33] and longer [34] lifespan among different groups of birds. *Region*) Immune investment varies with latitude [35], between tropical and non-tropical species [36], and between island and continental species [37], and parasite infection rates differ between continental regions [38]. I included species' native range in order to account for geographic differences in infection risk and immune investment.

The immune system is a complex trait and as such cannot be fully described by a single measure [39]. To date, few large comparative studies have used multiple immune measures, as it is logistically difficult to study at a broad phylogenetic and geographic scale. In this study, I assessed immune investment using leukocyte counts collected from captive individuals. Leukocytes are comprised of five cell types that function in both the innate and acquired immune responses [40]. Leukocytes have not been used in avian comparative studies, but are frequently used in mammalian studies where leukocyte counts are often explained by variables relating to disease risk, sexual selection and life history traits [28], [29], [41], [42], [43], [44].

I conducted this study on parrots (Psittaciformes). So far, passerines have been the focus of pace-of-life studies, and parrots are not often studied in the context of sexual selection though they are ideal for a number of reasons. Parrots are long-lived and have elaborate plumage characters. Because they are readily kept in captivity, particularly in zoos, comparative immune measures are available for a large number of species, and captive lifespan has been reported for many species [45]. The Psittaciformes are a relatively speciose group (approximately 375 species) but life history traits, such as diet and nesting location, are relatively invariable across species compared to passerines [46], [47]. That virtually all are cavity nesters of some sort means there is relaxed selection on female crypsis meaning that elaboration of plumage traits may more accurately reflect the strength of selection for those traits. Although there is little information available about the frequency of promiscuity in wild parrots, they are virtually all socially monogamous, forming and maintaining pair bonds during the breeding season [46]. As a result, hidden sources of sexual selection that may not explicitly be manifest as selection on plumage coloration are limited across species.

## Materials and Methods

### Leukocyte Data

I collected information for a total of 66 parrot species from 36 genera for which leukocyte data were available. I used baseline leukocyte values published in the International Species Information System (ISIS; Reference Ranges for Physiological Values in Captive Wildlife, 2002, Apple Valley, Minnesota Zoological Garden, MN, USA) as a measure of immunity. ISIS is a dataset of baseline haematological reference values taken from healthy captive animals from zoos and other housing institutions. Species mean values represent averages of data pooled from different member institutions (i.e. locations), and for both males and females. To investigate quality of the dataset I used coefficients of variation (CV) of total leukocyte counts. CV's were less than 1 (0.33–0.75) for all cases except for one (1.22) and so were taken to represent consistent counts across individuals and member institutions. Inclusion of the single species with high CV did not affect the results and so it was included in the analysis.

Plasma leukocyte counts increase for various reasons including in response to immunological challenge. However, ISIS data are collected only from generally healthy individuals to be used as part of a Complete Blood Count to assess sickness when monitoring captive animals. As such, counts from captive animals are likely less variable and may better reflect evolutionarily derived investment than measures from wild specimens.

One species was excluded from my analysis because it represented an extremely unique life history. *Nestor notabilis* is flightless, polygynous, and known to consume carrion. Flightlessness likely liberates energy for other physiological process such as immunity, and because it is ground-dwelling it is also likely constrained in the expression of bright plumage, which in this species is dull, despite facing high levels of sexual selection. Furthermore, increased disease transmission from scavenging carcasses may result in selection on immunity not related to sexual selection.

### Life-history Traits

Lifespan estimates (n = 56 for which leukocyte data were available) were taken from Brouwer *et al*. [45]. These estimates are the longest reported lifespans (years) for captive parrots, which can be interpreted as maximum potential lifespan (MLSP) for the species. Birds typically remain reproductively active until death [48], and intrinsic mortality in captivity reflects rates of intrinsic mortality in the wild [49]. For these reasons, traits associated with investment in longevity, such as immunity, are likely to relate to MLSP.

Life history traits were gathered though literature searches: body mass (g) was averaged over several sources ([46], [47], as well as from ISIS itself), sexual size dimorphism [50] and social group size [47], [50] was categorically defined as: small (2–14 birds), medium (15–50 birds), medium-large (50–200) and large (>200). All other life history traits (see table 1) were averaged across sources where possible [46], [47], [51] except for a small number of species where additional sources were needed [52], [53]. Sexual size dimorphism and dichromatism were categorized as absent, minimal or present. Diet was defined as: granivorous, frugivorous, generalist/omnivore and nectar feeding based on the majority composition of the diet. Habitat was categorized as either open, semi-open or closed and geographic region was based on the majority of the species natural range (grouped as America, Africa, Asia, Indonesia/Papua New Guinea/Malaysia and Australia). Life history traits are reported for wild individuals except for body mass.

**Table 1:** Life history parameters incorporated into five models used to explain leukocyte concentration.

	Sexual Selection	Pace-of-Life	Residual Pace-of-Life	Ecological	Global
Plumage Brightness	Y				Y
Sexual Dichromatism	Y				Y
Sexual Size Dimorphism	Y				Y
Incubation Period		Y	Y		Y
Clutch Size		Y	Y		Y
Longevity		Y	Y		Y
Log[Mass]			Y		Y
Diet				Y	Y
Region				Y	Y
Habitat				Y	Y
Flock Size				Y	Y

### Coloration

Several elements of plumage coloration and exaggeration of physical traits were ranked by five independent observers from painted colour plates [50]. Each species was scored from 1–5 (low to high) based on: i) Brightness - overall brightness of the plumage; ii) Contrast - the overall impression of the degree of contrasting plumage traits, iii) Complexity - the complexity of appearance including the number of colours and complexity of colour patterns including non-feather colour traits such as pigmented skin and feather adornments such as crests; iv) Non-background - the relative amount of non-background coloration could be suggestive of the strength of selection on traits that may not be otherwise bright or complex; v) The amount of Red/Orange/Yellow used in plumage traits. Where species were sexually dichromatic, observers were instructed to assess only male plumage coloration.

### Phylogeny

Phylogenetic hypotheses for parrots are relatively well established for higher taxonomic levels however species specific trees are either not available for a broad range of species or suffer discordances among studies. I used the phylogeny outlined by Mayr [54] which is derived from Wright *et al*. [55]) and includes modifications by other authors [56], [57]. This phylogeny encompasses all genera present in this study with the exception of *Psitteuteles*. Previous studies were informative [57], [58] but not effective for precisely placing Psitteuteles in my phylogeny so I determined the position of *Psitteuteles* relative to *Charmosyna*, *Lorius*, *Chalcopterus*, *Pseudeos*, *Eos* and *Trichoglossus* by reconstructing the clade using a bayesian Markov Chain Monte Carlo approach implemented in MrBayes [59]. Cytochrome b sequences were obtained from Genbank [60] and aligned in BioEdit [61] resulting in a shared sequence 853 bp in length. I used a general time reversible Bayesian model run for 10,000 generations and sampled every 10 generations following a burn in of 2000 generations. The resulting tree structure placed *Psitteuteles* in its present position in the phylogeny (electronic supplementary material, figure S1) with a posterior probability of 100%.

The phylogeny was further modified to resolve species relationships for the speciose clades for which I had immunological data for by appending known phylogenies for *Chalcopsitta* [62], *Amazona* [63] and *Cacatua* [64]. Since no phylogeny exists for *Ara*, I constructed one from a 430 bp shared fragment of the ribosomal RNA 16 s gene (electronic supplementary material, figure S2) following the same Bayesian procedure used for *Psitteuteles* but using a burn in of 1600 generations.

### Statistical Analyses

All statistical analyses were done using R 2.11 [65]. I assessed model fit using AIC values corrected for small samples sizes [66] derived from phylogenetically informed Generalized Least Squares (PGLS) regression models using the R packages nlme [67] and Ape [68]. From the parameters measured I identified 5 models comprising a sexual selection model, a pace-of-life model, residual pace-of-life (accounting for body mass), an ecological model and a global model (table 1). For each statistical model I compared the fit of two evolutionary models. PGLS λ transforms branch lengths by optimizing their length based on trait evolution determined by the phylogeny [69]. The λ parameter varies between 0 (phylogenetic independence) and 1 (phylogenetic dependence). The Ornstein-Uhlenbeck process applies a similar branch length transformation however assumes a constraint on trait evolution [70]. Results from these models were similar and did not influence their interpretation, and so all results given are from PGLS λ models. I used the first principal component of all plumage variables as a measure of plumage exaggeration (table 2). I used phylogenetic PC [71] scores obtained using the λ method because using PC scores derived using λ and brownian motion models did not influence the interpretation of the results.

**Table 2:** Eigenvectors for plumage traits from a phylogenetically informed PCA.

Plumage Trait	PC1
Brightness	0.82
Contrast	0.85
Complexity	0.70
Non-background	0.80
Non-green	0.51
Red/Orange/Yellow	0.88

## Results

The ISIS dataset included 66 species of parrot ranging from 3–119 individuals per species (median: 12 individuals per species). Across species, heterophils comprised 53% of all leukocytes followed by lymphocytes (37%), monocytes (5%), eosinophils (3%) and basophils (2%). Initial investigation revealed two outliers (>3.6 standard deviations) for total leukocyte counts. The cause of this was extremely high heterophil counts (4.5 and 5.5 standard deviations above mean counts). These outliers had a strong effect on subsequent models and were identified as highly influential based on Cook's distance values derived from residual-leverage plots. These two species were two of three species from the genus *Chalcopsitta*. The third species also had very high leukocyte counts but were within the range expected for all species. However, its high leukocytes were again driven by extremely high heterophil counts (3.4 standard deviations). Because all members of *Chalcopsitta* in this study show these extreme counts they appear to be evolutionarily derived but it is not clear what aspect of their life history accounts for selection for such high counts. Because of the influence of these extreme data points all three were excluded from analyses involving heterophils, and the former two were excluded from analyses of leukocytes.

Correlations among the five leukocyte types were generally low. The strongest relationships were between heterophils and eosinophils (r = 0.29, P = 0.02) and between lymphocytes and monocytes (r = 0.36, P = 0.004), which represents natural groupings of granulocytic and non-granulocytic leukocytes. All cell types increased with total leukocyte count, but the strength of the relationship was proportional to the abundance of the cell type.

Species with more exaggerated plumage traits had higher leukocyte counts (table 3). This was especially the case for overall brightness and the extent of red, orange and yellow based plumage, and to a lesser extent the degree of contrast. On the other hand, aspects complexity, including the amount of non-background coloration was not related to leukocytes. Among leukocyte types, heterophils, lymphocytes and basophils were positively related to plumage colour but eosinophils and monocytes were not related (table 3).

**Table 3:** The relationship between aspects of leukocytes and plumage coloration from bivariate analyses.

Predictor	Trait	n	λ	AICc	t	P
Total Leukocytes	PC Plumage	64	0.05^ns,*^	310.58	2.67	**0.01**
	Red/Orange/Yellow	64	0.04^ns,*^	303.46	3.61	**<0.001**
	Brightness	64	0.04^ns,*^	303.56	3.32	**0.002**
	Contrast	64	0.02^ns,*^	309.73	2.04	**0.05**
	Complexity	64	0.17^ns,*^	311.81	0.91	0.37
	Non-background	64	0.18^ns,*^	317.81	1.05	0.30
	Non-green	64	0.17^ns,*^	318.58	1.24	0.22
Heterophils	PC Plumage	63	0.41^*,*^	259.56	2.22	**0.03**
Lymphocytes	PC Plumage	64	0.50^*,*^	235.77	2.38	**0.02**
Eosinophils	PC Plumage	63	0.02^ns,*^	−2.64	−0.24	0.81
Monocytes	PC Plumage	64	0.18^ns,*^	11.19	−0.20	0.84
Basophils	PC Plumage	64	0.10^ns,*^	−84.52	2.24	**0.03**

In general, species with more elaborate, but not more complex, plumage had higher leukocyte counts. λ values were tested against null models (λ = 0, complete phylogenetic independence) or phylogenetic dependence (λ = 1) using likelihood ratio tests. Significant differences from 0 (first position) and 1 (second position) are denoted by *. Eosinophil counts were not available for one species. See Results for sample size explanations otherwise.

Some data were not available for all species and so the comparison of models presented in table 1 were done on a reduced dataset (n = 45). The sexual selection model performed the best of all models (table 4). PC plumage colour (t = 3.09, P = 0.004) was positively related to leukocyte count whereas sexual dichromatism was negatively related (t = −3.28, P = 0.002). The degree of sexual size dimorphism was not related to leukocytes. The remaining models achieved relatively little support. Individual variables included in those models were poor predictors of leukocyte counts with a couple of exceptions. In the ecological model, species associating in larger flocks had fewer leukocytes (t = −2.38, P = 0.02; all other variables P>0.17). Generally, pace-of-life models performed poorly but the length of the incubation period was positively related to leukocytes (residual pace-of-life model: t = 2.30, P = 0.03; pace-of-life model: t = 2.45, P = 0.02; all other variables P>0.66 for both models). Plumage colour (t = 3.03, P = 0.005) was the only variable that remained significant in the in the global model although flock size (t = −1.79, P = 0.08) and incubation period (t = 1.79, P = 0.08) approached significance.

**Table 4:** Performance of selected models explaining total leukocyte counts.

Model	λ	K	AICc	ΔAICc	*L*ΔAICc	AICc*W*	Evidence Ratio
Sexual Selection	−0.16^ns,*^	3	207.387	0.000	1.000	0.991	1.000
Ecological	−0.16^ns,*^	4	216.877	9.490	0.009	0.009	115.033
Pace-of-Life	0.24^ns,*^	3	222.955	15.568	0.000	0.000	2402.103
Residual POL	0.25^*,*^	4	236.316	28.929	0.000	0.000	1913739.990
Global	0.15^ns,*^	11	239.387	32.000	0.000	0.000	8887621.288

Models were run with a subset of species (n = 45) for which all data were available.

## Discussion

### Stronger Sexual Selection has both a positive and negative effect on Immune Investment

Plumage colour was positively related to leukocyte counts and was the strongest single variable predicting leukocyte counts in any model. Similarly, the sexual selection model performed better than the pace-of-life and ecological models. Although I was not able to compare infection rates in this study, others have shown that brighter species tend to have higher rates of infection ([17], [25], [72], but see [73]). These findings reinforce the idea that selection for brighter, more immuno-competent individuals within species results in greater species level immune investment. Moreover, bearing these elaborate traits does not result in a cost imposed on the immune system as could result from an immuno-competence handicap type mechanism [14].

By contrast, immune investment was lower in species with marked sexual dichromatism. This is consistent with studies showing greater mortality in dichromatic species [11], but is contrary to studies demonstrating a positive relationship between dichromatism and spleen size [74], [75]. Cavity nesting, which occurs in all parrots in some form [46], liberates incubating females from selection on crypsis and so sexual dichromatism is less frequent in cavity nesting species [76]. This means that expression of plumage coloration can be similar in males and females with species brightness reflecting selection for, but not constraint on, plumage coloration. Sexual dichromatism more frequently occurs due to selection for reduced ornamentation in one sex [77]. Yet, there is no obvious cause for dichromatism in the species of parrots in my sample. Female crypsis should occur in species where nests are more exposed but dichromatic species in this study do not use more open nesting hollows versus cavities nor is there an interaction with plumage brightness.

Sexual size dimorphism was not related to leukocytes. However, size dimorphism may not be an accurate measure if it is more detectable in larger species. If this is the case, it would result in biased estimates of the degree of dimorphism when grouping birds across a large range of body sizes. I addressed this by analyzing those species less than 120 g, and separately only large bodied species (>415 g), and there was still no effect of sexual size dimorphism on leukocytes in either case. In most birds, sexual size dimorphism results from divergent sex roles [78], but parrots are largely monogamous, with males feeding females throughout the incubation period. This likely explains why sexual size dimorphism is limited in parrots, with fewer than 40% of the species in this study exhibiting some level of size dimorphism.

It would be insightful to understand the interplay between sexual size dimorphism, sexual dichromatism and plumage coloration. However, it is clear that as a measure of the intensity of sexual selection, sexual size dimorphism does not explain investment in the immune system in parrots.

Taken together, leukocyte counts were positively related to overall plumage expression, yet negatively related to the degree of sexual dichromatism. Thus, absolute trait expression and the degree of difference in trait expression between the sexes had opposing effects on immunity. Costs associated with bearing elaborate SSC's, such as reduced immunity and/or higher mortality across species, may not be a function of the trait per sé but rather behaviours associated with large sexual dimorphisms since sexual dimorphisms result from different selective trajectories for males and females [18]. For example, higher costs in more dimorphic species [9], [10], [11], [44] may stem from behaviours associated with more elaborate sexual traits and divergent behaviours such as courtship, territoriality, or other energetic trade-offs independent of specific costs of the traits being measured.

### Longevity, Pace-of-life and Immunity

Both longevity and residual longevity proved to be surprisingly poor predictors of investment in immunity. Lifespan and immunity are expected to be positively related either because a competent immune system enables long life, or because species with genetically determined long lives preferentially invest in immunity to achieve their maximum lifespan potential. In the few cases where lifespan has been used in comparative studies, it was not related to cell-mediated immunity in birds [21], but was related to leukocyte counts in mammals, although the result was limited to females [44]. As expected though, longevity was predictably correlated with pace-of-life traits (incubation period r = 0.41, P = 0.005; clutch size r = −0.38, P = 0.009) and the relationship between body size and longevity was strong (r = 0.52, P<0.001) and consistent with what is typically reported in birds [79], [80].

Some have argued that MLSP is not as biologically relevant as longevity in the wild ([81], but see [49]) possibly because specific traits, such as those associated with pace-of-life, are likely the proximate determinants of trade-offs with other life history traits, with shorter MLSP simply being a by-product of increased emphasis on reproduction. In the present study, pace-of-life models received relatively little empirical support based on AIC values, however, incubation period was positively related to leukocyte counts. This result is consistent with pace-of-life studies, which frequently show relationships between immune investment and incubation period but not with other pace-of-life traits, such as clutch size [21], [23], [24].

Species with long incubation periods typically have young that take longer to fledge and generally slower developmental rates. Longer developmental periods in the nest inherently expose offspring to an increased risk of nest-born parasites, something that could be especially problematic for cavity nesting species that tend to reuse nest sites. Alternatively, slow development may delay maturation of the acquired immune response which increases selection on innate aspects of immunity [39]. This may be the case because among the pace-of-life variables used in the present study, only incubation period was related to leukocyte counts. On the other hand, increased maternal antibody transfer does not seem adequate to offset the delayed maturation of the acquired immune system in slow paced species [82], although it has been suggested that persistence of these antibodies might be greater in species with slower pace-of-life [83].

### More gregarious species have fewer leukocytes

Generally, the ecological model did not explain immune investment. However, I found that parrot species that form larger flocks have fewer leukocytes. This seems counterintuitive since the risk of infection should increase at higher densities [26] and in support of this, immune investment is often higher among animals in larger groups [27], [28], [29]. Yet several studies, including this one, report the opposite pattern (e.g., [24], [84]), which has led to the hypothesis that in large groups, individuals may benefit from a host dilution effect [85], [86]. This may be an appropriate explanation for parrots because flocks are often temporally or spatially ephemeral [46] which may limit the ability for vectors to track host aggregations.

### A note on Red-, Orange- and Yellow-based Coloration in Parrots

Most plumage traits were positively correlated amongst themselves but brightness, contrast and red/orange/yellow were the most important variables comprising the first principal component of plumage colour (table 2). Not surprisingly, the degree of red, orange and yellow coloration was strongly related to leukocyte count. Unlike in passerines where these colours are produced by dietary carotenoids, in parrots the colours result from a less studied class of non-dietary pigments called the psittacofulvins [87]. The present study evidences a link between the immune system and psittacofulvins. Psittacofulvin-derived plumage traits have been shown to be condition dependent [88], and psittacofulvins themselves have antioxidant properties [89], [90], and so likely signal similar physiological information to carotenoid-based traits. However, it is noteworthy that parrots use psittacolfulvins in lieu of carotenoids despite having adequate plasma carotenoid levels [91]. Understanding the relative costs and the contributions to physiological processes of these two pigments is an interesting avenue for future research.

The complexity of the immune system cannot be adequately described using a single measure, and because of this the results from this study should be considered accordingly. Inconsistencies across comparative studies can be attributed, in part, to individual immune measures being related to life history traits in unique ways. I used leukocyte counts as a means of assessing immune investment. Although this measure has been considered unreliable for wild species [92], circulating leukocytes are regularly used to assess health and immunocompetence in both captive animals and humans.

### Conclusion

The current comparative study adds to our knowledge of the role the immune system plays in the relationship between plumage brightness and parasite loads. Arguably, high parasite loads in brighter species could be the result of poor investment in immunity, perhaps as a consequence of investment in costly SSC's or other aspects of reproduction. By contrast, the results from the present study are consistent with intra-sexual selection producing brighter and more immuno-competent individuals, and that elaborate plumage need not be a handicap as proposed by the ICHH. I did note a negative effect of sexual dichromatism, which suggests the need to control for divergent sexual strategies when studying the costliness of SSC's.

Similar to other recent studies [21], [44], longer lived species did not invest more in leukocytes, however species with slower pace-of-life did. Pace-of-life traits may be a better measure of immune investment than longevity, and in particular, immune investment may be related costs associated with long developmental periods.

## Supporting Information

Figure S1 **Phylogeny of the 66 parrot species for which there were leukocyte data.** The tree is that of Mayr (2010) with modifications from several other sources (see Material and Methods).
(TIFF)

Figure S2 **Bayesian tree constructed from a 430 bp RNA 16 s gene fragment for the genus** ***Ara*** **.** Posterior probabilities evidence strong support for most branches in the tree. *P. erithacus* and *A. hyacinthinus* were used as outgroups.
(TIFF)

## References

[1] Lochmiller RL, Deerenberg C (2000) Trade-offs in evolutionary immunology: Just what is the cost of immunity? Oikos 88: 87–98 (doi:10.1034/j.1600-0706.2000.880110.x)

[2] Norris K, Evans MR (2000) Ecological immunology: life history trade-offs and immune defense in birds. Behav Ecol 11: 19–26 (doi:10.1093/beheco/11.1.19)

[3] Williams GC (1966) Natural selection, the costs of reproduction, and a refinement of Lack's principle. Am Nat 100: 687–690 (doi:10.1086/282461)

[4] Stearns SC (1992) The evolution of life histories. Oxford,UK: Oxford University Press. 262p.

[5] Råberg L, Nilsson J, Ilmonen P, Stjernman M, Hasselquist D (2000) The cost of an immune response: vaccination reduces parental effort. Ecol Lett 3: 382–386 (doi:10.1046/j.1461-0248.2000.00154.x)

[6] Martin LB, Scheuerlein A, Wikelski M (2003) Immune activity elevates energy expenditure of house sparrows: a link between direct and indirect costs? ProcBiol Sci 270: 153–158 (doi:10.1098/rspb.2002.2185) 10.1098/rspb.2002.2185 PMC1691219 12590753

[7] Sheldon BC, Verhulst S (1996) Ecological immunology: costly parasite defences and trade-offs in evolutionary ecology. Trends Ecol Evol 11: 317–321 (doi:10.1016/0169-5347(96)10039-2) 21237861 10.1016/0169-5347(96)10039-2

[8] Zuk M, Stoehr AM (2002) Immune defence and host life history. Am Nat 160: S9–S22 (doi: 10.1086/342131) 18707455 10.1086/342131

[9] Promislow DEL (1992) Costs of sexual selection in natural populations of mammals. Proc Biol Sci 247: 203–210 (doi:10.1098/rspb.1992.0030)

[10] Moore SL, Wilson K (2002) Parasites as a viability cost of sexual selection in natural populations of mammals. Science 297: 2015–2018 (doi:10.1126/science.1074196) 12242433 10.1126/science.1074196

[11] Promislow DEL, Montgomerie R, Martin TE (1992) Mortality costs of sexual dimorphism in birds. Proc Biol Sci 250: 143–150 (doi:10.1098/rspb.1992.0142)

[12] Promislow DEL, Montgomerie R, Martin TE (1994) Sexual selection and survival in North American waterfowl. Evolution 48: 2045–2050 (doi:10.2307/2410526) 28565159 10.1111/j.1558-5646.1994.tb02232.x

[13] Zahavi A (1975) Mate selection: a selection for a handicap. J Theor Biol 53: 205–214 (doi:10.1016/0022-5193(75)90111-3) 1195756 10.1016/0022-5193(75)90111-3

[14] Folstad I, Karter AJ (1992) Parasites, bright males, and the immunocompetence handicap. Am Nat 139: 603–622 (doi:10.1086/285346)

[15] Andersson M (1994) Sexual Selection. Princeton: Princeton University Press. 624p.

[16] Møller AP, Christe P, Lux E (1999) Parasitism, host immune function and sexual selection. Q Rev Biol 74: 3–20 (doi:10.1086/392949) 10081812 10.1086/392949

[17] Hamilton WD, Zuk M (1982) Heritable true fitness and bright birds: a role for parasites? Science 218: 384–387 (doi:10.1126/science.7123238) 7123238 10.1126/science.7123238

[18] Arnqvist G, Rowe L (2005) Sexual Conflict. Princeton: Princeton University Press. 330p.

[19] Promislow DEL, Harvey PH (1990) Living fast and dying young: A comparative analysis of life-history variation among mammals. J Zool (1987) 220: 417–437 (doi:10.1111/j.1469-7998.1990.tb04316.x)

[20] Bonduriansky R, Maklakov A, Zajitschek F, Brooks R (2008) Sexual selection, sexual conflict and the evolution of ageing and life span. Funct Ecol 22: 443–453 (doi:10.1111/j.1365-2435.2008.01417.x)

[21] Tella JL, Scheuerlein A, Ricklefs RE (2002) Is cell-mediated immunity related to the evolution of life-history strategies in birds? Proc Biol Sci 269: 1059–1066 (doi:10.1098/rspb.2001.1951) 12028764 10.1098/rspb.2001.1951 PMC1690995

[22] Tieleman BI, Williams JB, Ricklefs RE, Klasing KC (2005) Constitutive innate immunity is a component of the pace-of-life syndrome in tropical birds. Proc Biol Sci 272: 1715–1720 (doi:10.1098/rspb.2005.3155) 16087427 10.1098/rspb.2005.3155 PMC1559858

[23] Palacios MG, Martin TE (2006) Incubation period and immune function: a comparative field study among coexisting birds. Oecologia 146: 505–512 (doi:10.1007/s00442-005-0220-3) 16217680 10.1007/s00442-005-0220-3

[24] Lee KA, Wikelski M, Robinson WD, Robinson TR, Klasing KC (2008) Constitutive immune defences correlate with life-history variables in tropical birds. J Anim Ecol 77: 356–363 (doi:10.1111/j.1365-2656.2007.01347.x) 18194261 10.1111/j.1365-2656.2007.01347.x

[25] Read AF (1987) Comparative evidence supports the Hamilton and Zuk hypothesis on parasites and sexual selection. Nature 328: 68–70 (doi:10.1038é328068a0)

[26] Côté IM, Poulin R (1995) Parasitism and group size in animals: a meta-analysis. Behav Ecol 6: 159–165 (doi:10.1016/j.aquatox.2009.03.002)

[27] Møller AP, Merino S, Brown CR, Robertson RJ (2001) Immune defense and host sociality: A comparative study of swallows and martins. Am Nat 158: 136–145 (doi:10.1086/321308) 18707342 10.1086/321308

[28] Nunn CL, Gittleman JL, Antonovics J (2003) A comparative study of white blood cell counts and disease risk in carnivores. Proc Biol Sci 270: 347–356 (doi:10.1098/rspb.2002.2249) 12639313 10.1098/rspb.2002.2249 PMC1691255

[29] Nunn CL (2002) A comparative study of leukocyte counts and disease risk in primates. Evolution 56: 177–190 (doi:10.1554/0014-3820(2002)056[0177:ACSOLC]2.0.CO;2). 11913662 10.1111/j.0014-3820.2002.tb00859.x

[30] Endler JA (1993) The color of light in forests and its implications. Ecol Monogr 63: 1–27 (doi:10.2307/2937121)

[31] McNaught MK, Owens IPF (2002) Interspecific variation in plumage colour among birds: species recognition or light environment. J Evol Biol 15: 505–514 (doi:10.1046/j.1420-9101.2002.00431.x)

[32] Catoni C, Schaefer HM, Peters A (2008) Fruit for health: the effect of flavonoids on humoral immune response and food selection in a frugivorous bird. Funct Ecol 22: 649–654 (doi:10.1111/j.1365-2435.2008.01400.x)

[33] Peach WJ, Hammer DB, Oatley TB (2001) Do southern African songbirds live longer than their European counterparts? Oikos 93: 235–249 (doi:10.1034/j.1600-0706.2001.930207.x)

[34] Munshi-South J, Wilkinson GS (2006) Diet influences life span in parrots (Psittaciformes). Auk 123: 108–118 (doi:10.1642/0004-8038(2006)123[0108:DILSIP]2.0.CO;2).

[35] Møller AP (1998) Host immune defence and migration in birds. Evol Ecol 12: 945–953 (doi:10.1023/A:1006516222343)

[36] Møller AP, Erritzøe J (1998) Evidence of larger impact of parasites on hosts in the tropics: investment in immune function within and outside the tropics. Oikos 82: 265–270 (doi:10.2307/3546966)

[37] Matson KD (2006) Are there differences in immune function between continental and insular birds? Proc Biol Sci 273: 2267–2274 (doi:10.1098/rspb.2006.3590) 16928627 10.1098/rspb.2006.3590 PMC1636094

[38] Valkiunas G (2004) Avian malaria parasites and other haemosporidia. Boca Raton: CRC Press, 932 p.

[39] Lee KA (2006) Linking immune defenses and life history at the levels of the individual and the species. Integr Comp Biol 46: 1000–1015 (doi:10.1093/icb/icl049) 21672803 10.1093/icb/icl049

[40] Roitt I, Brostoff J, Male D (eds) (1993) Immunology, 3^rd^ ed. London: Mosby. 420p.

[41] Nunn CL, Gittleman JL, Antonovics J (2000) Promiscuity and the primate immune system. Science 290: 1168–1170 (doi:10.1126/science.290.5494.1168) 11073457 10.1126/science.290.5494.1168

[42] Semple S, Colishaw G, Bennett PM (2002) Immune system evolution among anthropoid primates: parasites, injuries and predators. Proc Biol Sci 269: 1031–1037 (doi:10.1098/rspb.2001.1950) 12028760 10.1098/rspb.2001.1950 PMC1690991

[43] Anderson MJ, Hessel JK, Dixson AF (2004) Primate mating systems and the evolution of immune responses. J Reprod Immunol 61: 31–38 (doi:10.1016/j.jri.2003.11.001) 15027476 10.1016/j.jri.2003.11.001

[44] Nunn CL, Lindenfors P, Pursall ER, Rolff J (2009) On sexual dimorphism in immune function. Philos Trans R Soc Lond B Biol Sci 364: 61–69 (doi: doi:10.1098/rstb.2008.0148) 18926977 10.1098/rstb.2008.0148 PMC2666693

[45] Brouwer K, Jones ML, King CE, Schifter H (2000) Longevity records for Psittaciformes in captivity. International Zoo Yearbook 37: 299–316.

[46] del Hoyo J, Elliot A, Sargatal J (eds) (1997) Handbook of the Birds of the World. Volume 4: Sandgrouse to Cuckoos. Barcelona: Lynx Edicions. 600p.

[47] Juniper T, Parr M (1998) Parrots: a guide to parrots of the world. New Haven: Yale University Press. 584 p.

[48] Holmes DJ, Austad SN (1995) The evolution of avian senescence patterns: implications for understanding primary aging processes. Am Zool 35: 307–317 (doi:10.1093/icb/35.4.307)

[49] Ricklefs RE (2000) Intrinsic aging-related mortality in birds. J Avian Biol 31: 103–111 (doi:10.1034/j.1600-048X.2000.210201.x)

[50] Forshaw JM (1973) Parrots of the World. New York: Doubleday. 584 p.

[51] Dunning JB (2008) CRC handbook of avian body masses, 2^nd^ ed. Boca Raton: Taylor and Francis Group. 672 p.

[52] Low R (1998) Encyclopedia of Lories. Surrey: Hancock House. 432 p.

[53] Alderton D (2000) The Ultimate Encyclopedia of Caged and Aviary Birds. New York: Lorenz Books. 256 p.

[54] Mayr G (2010) Parrot interrelationships – morphology and the new molecular phylogenies. Emu 110: 348–357 (doi:10.1071/MU10035)

[55] Wright TF, Schirtzinger EE, Matsumoto T, Eberhard JR, Graves GR, et al (2008) A multilocus molecular phylogeny of the parrots (Psittaciformes): support for a Gondwanan origin during the Cretaceous. Mol Biol Evol 25: 2141–2156 (doi:10.1093/molbev/msn160) 18653733 10.1093/molbev/msn160 PMC2727385

[56] Tavares ES, Baker AJ, Pereira SL, Miyaki CY (2006) Phylogenetic relationships and historical biogeography of Neotropical Parrots (Psittaciformes: Psittacidae: Arini) inferred from mitochondrial and nuclear DNA sequences. Syst Biol 55: 454–470 (doi:10.1080/10635150600697390) 16861209 10.1080/10635150600697390

[57] Schweizer M, Sechausen O, Güntert M, Hertwig ST (2010) The evolutionary diversification of parrots supports a taxon pulse model with multiple trans-oceanic dispersal events and local radiations. Mol Phylogenet Evol 54: 984–994 (doi:10.1016/j.ympev.2009.08.021) 19699808 10.1016/j.ympev.2009.08.021

[58] Christidis L, Schodde R, Shaw DD, Maynes SF (1991) Relationships among the Australo-Papuan Parrots, Lorikeets and Cockatoos (Aves: Psittaciformes): Protein evidence. Condor 93: 302–317 (doi:10.2307/1368946)

[59] Huelsenbeck JP, Ronquist F (2001) MRBAYES: Bayesian inference of phylogeny. Bioinformatics 17: 754–755 (doi:10.1093/bioinformatics/17.8.754) 11524383 10.1093/bioinformatics/17.8.754

[60] Benson DA, Karsch-Mizrachi I, Lipman DJ, Ostell J, Wheeler DL (2005) GenBank. Nucleic Acids Res 33: D34–D38 (doi:10.1093/nar/gki063) 15608212 10.1093/nar/gki063 PMC540017

[61] Hall TA (1999) BioEdit: a user-friendly biological sequence alignment editor and analysis program for Windows 95/98/NT. Nucleic Acids Symposium Series 41: 95–98.

[62] Astuti D, Aruma N, Suzuki H, Higashi S (2006) Phylogenetic relationships within parrots (Psittacidae) inferred from mitochondrial Cytochrome-b gene sequences. Zoolog Sci 23: 191–198 (doi:10.2108ézsj.23.191) 16603811 10.2108/zsj.23.191

[63] Russello MA, Amato G (2004) A molecular phylogeny of Amazona: implications for Neotropical parrot biogeography, taxonomy, and conservation. Mol Phylogenet Evol 30: 421–437 (doi:10.1016/S1055-7903(03)00192-1) 14715233 10.1016/s1055-7903(03)00192-1

[64] Brown DM, Toft CA (1999) Molecular systematics and biogeography of the cockatoos (Psittaciformes: Cacatuidae). Auk 116: 141–157.

[65] R Development Core Team (2008) R: A language and environment for statistical computing. R Foundation for Statistical Computing, Vienna, Austria. ISBN 3-900051-07-0, URL http://www.R-project.org.

[66] Burnham KP, Anderson DR (2002) Model selection and mulitmodel inference: A practical information-theoretic approach. New York: Springer Verlag. 488 p.

[67] Pinheiro J, Bates D, DebRoy S, Sarkar D, the R Development Core Team (2011) nlme: Linear and Nonlinear Mixed Effects Models. R package version 3.1-98.

[68] Paradis E, Claude J, Strimmer K (2004) APE: analysis of phylogenetics and evolution in R language. Bioinformatics 20: 289–290 (doi:10.1093/bioinformatics/btg412) 14734327 10.1093/bioinformatics/btg412

[69] Pagel M (1999) Inferring the historical patterns of biological evolution. Nature 401: 877–884 (doi:10.1038/44766) 10553904 10.1038/44766

[70] Martins EP, Hansen TF (1997) Phylogenies and the comparative method: a general approach to incorporating phylogenetic information into the analysis of interspecific data. Am Nat 149: 646–667 (doi:10.1086/303188)

[71] Revell LJ (2012) phytools: An R package for phylogenetic comparative biology (and other things). Methods Ecol Evol 3: 217–223 (doi:10.1111/j.2041-210X.2011.00169.x)

[72] Scheuerlein A, Ricklefs RE (2004) Prevalence of blood parasites in European passeriform birds. Proc Biol Sci 271: 1363–1370 (doi:10.1098/rspb.2004.2726) 15306334 10.1098/rspb.2004.2726 PMC1691737

[73] Read AF, Harvey PH (1989) Reassessment of comparative evidence for Hamilton and Zuk theory on the evolution of secondary sexual characters. Nature 339: 18–620 (doi:10.1038/339618a0)

[74] Møller AP (1997) Immune defence, extra-pair paternity, and sexual selection. Proc Biol Sci 264: 561–566 (doi:10.1098/rspb.1997.0080)

[75] Møller AP, Dufva R, Erritzøe J (1998) Host immune function and sexual selection in birds. J Evol Biol 11: 703–719.

[76] Scott DK, Clutton-Brock TH (1990) Mating systems, parasites and plumage dimorphism in waterfowl. Behav Ecol Sociobiol 26: 261–273 (doi: 10.1007/BF00178319)

[77] Badyaev AV, Hill GE (2003) Avian sexual dichromatism in relation to phylogeny and ecology. Annu Rev Ecol Evol Syst 34: 27–49 (doi: 10.1146/annurev.ecolsys.34.011802.132441)

[78] Fairbairn DJ, Blanckenhorn, Székely T (2007) Sex, size and gender roles: Evolutionary studies of sexual size dimorphism. Oxford: Oxford University Press Online. (doi: 10.1093/acprof:oso/9780199208784.001.0001)

[79] Hulbert AJ, Pamplona R, Buffenstein R, Buttemer WA (2007) Life and death: metabolic rate, membrane composition and life span of animals. Physiol Rev 87: 1175–1213 (doi:10.1152/physrev.00047.2006) 17928583 10.1152/physrev.00047.2006

[80] de Magalhaes JP, Costa J, Church GM (2007) An analysis of the relationship between metabolism, developmental schedules, and longevity using phylogenetic independent contrasts. J Gerontol 62A: 149–160. 10.1093/gerona/62.2.149 PMC2288695 17339640

[81] Monoghan P, Metcalfe NB (2001) Genome size, longevity and development time in birds. Trends Genet 17: 568 (doi:10.1016/S0168-9525(01)02415-5) 10.1016/s0168-9525(01)02414-3 11642250

[82] Addison B, Klasing KC, Robinson WD, Austin SH, Ricklefs RE (2009) Ecological and life-history factors influencing the evolution of maternal antibody allocation: a phylogenetic comparison. Proc Biol Sci 276: 3979–3987 (doi: 10.1098/rspb.2009.1296) 19710063 10.1098/rspb.2009.1296 PMC2825792

[83] Garnier R, Ramos R, Staszewski V, Militao T, Lobato E, et al (2012) Maternal antibody persistence: a neglected life-history trait with implications from albatross conservation to comparative immunology. Proc Biol Sci 279: 2033–2041 (doi: 10.1098/rspb.2011.2277) 22189405 10.1098/rspb.2011.2277 PMC3311891

[84] Watve MG, Sukumar R (1995) Parasite abundance and diversity in mammals correlates with host ecology. Proc Natl Acad Sci USA 92: 8945–8949. 7568049 10.1073/pnas.92.19.8945 PMC41084

[85] Wilson K, Knell R, Boots M, Koch-Osborne J (2003) Group living and investment in immune defence: an interspecific analysis. J Anim Ecol 72: 133–143.

[86] Watve MG, Jog MM (1997) Epidemic diseases and host clustering: an optimum cluster size ensures maximum survival. J Theor Biol 1: 167–171 (doi: 10.1006/jtbi.1996.0267) 10.1006/jtbi.1996.0267 9059596

[87] Stradi R, Pini E, Celentano G (2001) The chemical structure of the pigments in *Ara macao* plumage. Comp Biochem Physiol B Biochem Mol Biol 130: 57–63 (doi:10.1016/S1096-4959(01)00402-X) 11470444 10.1016/s1096-4959(01)00402-x

[88] Masello JF, Lubjuhn T, Quillfeldt P (2008) Is the structural and psittacofulvin-based coloration of wild burrowing parrots *Cyanoliseus patagonus* condition dependent? J Avian Biol 39: 653–662 (doi:10.1111/j.1600-048X.2008.04417.x)

[89] Morelli R, Loscalzo R, Stradi R, Bertelli A, Falchi M (2003) Evaluation of the antioxidant activity of new carotenoid-like compounds by electron paramagnetic resonance. Drugs Exp Clin Res 29: 95–100. 14708454

[90] Pini E, Bertelli A, Stradi R, Falchi M (2004) Biological activity of parrodienes, a new class of polyunsaturated linear aldehydes similar to carotenoids. Drugs Exp Clin Res 30: 203–206. 15700747

[91] McGraw KJ, Nogare MC (2005) Distribution of unique red feather pigments in parrots. Biol Lett 1: 38–43. 17148123 10.1098/rsbl.2004.0269 PMC1629064

[92] Hasselquist D (2007) Comparative immunoecology in birds: hypotheses and tests. J Ornithol 148: S571–S582.

