# Supplementary figures and images for: Immune Investment Is Explained by Sexual Selection and Pace-of-Life, but Not Longevity in Parrots (Psittaciformes)
Source: PLoS One. 2012 Dec 27;7(12):e53066. doi: 10.1371/journal.pone.0053066 (PMC3531452; doi:10.1371/journal.pone.0053066)

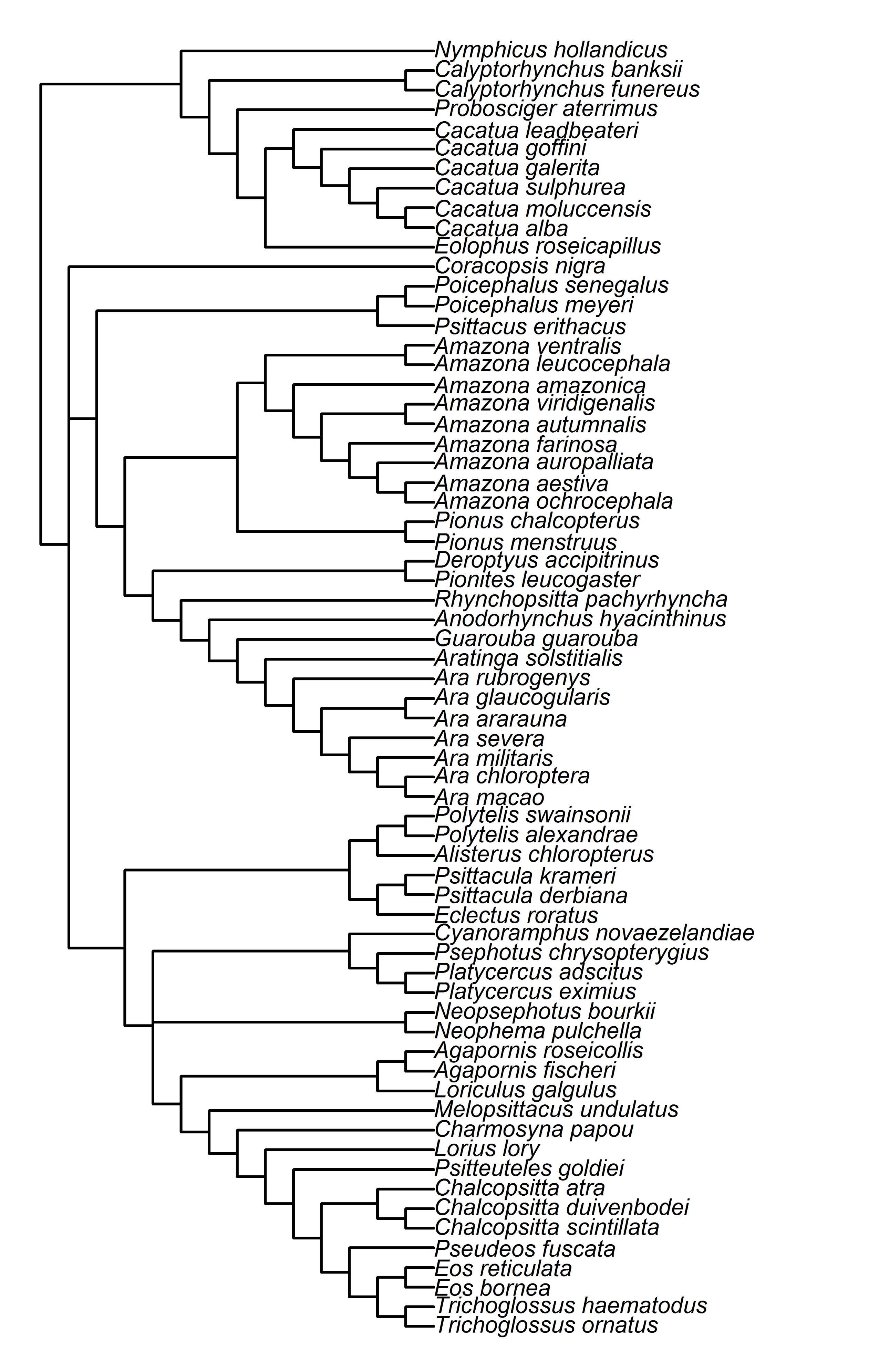

Supplement: Figure S1 — Phylogeny of the 66 parrot species for which there were leukocyte data. The tree is that of Mayr (2010) with modifications from several other sources (see Material and Methods). (TIFF) [file pone.0053066.s001.tiff]

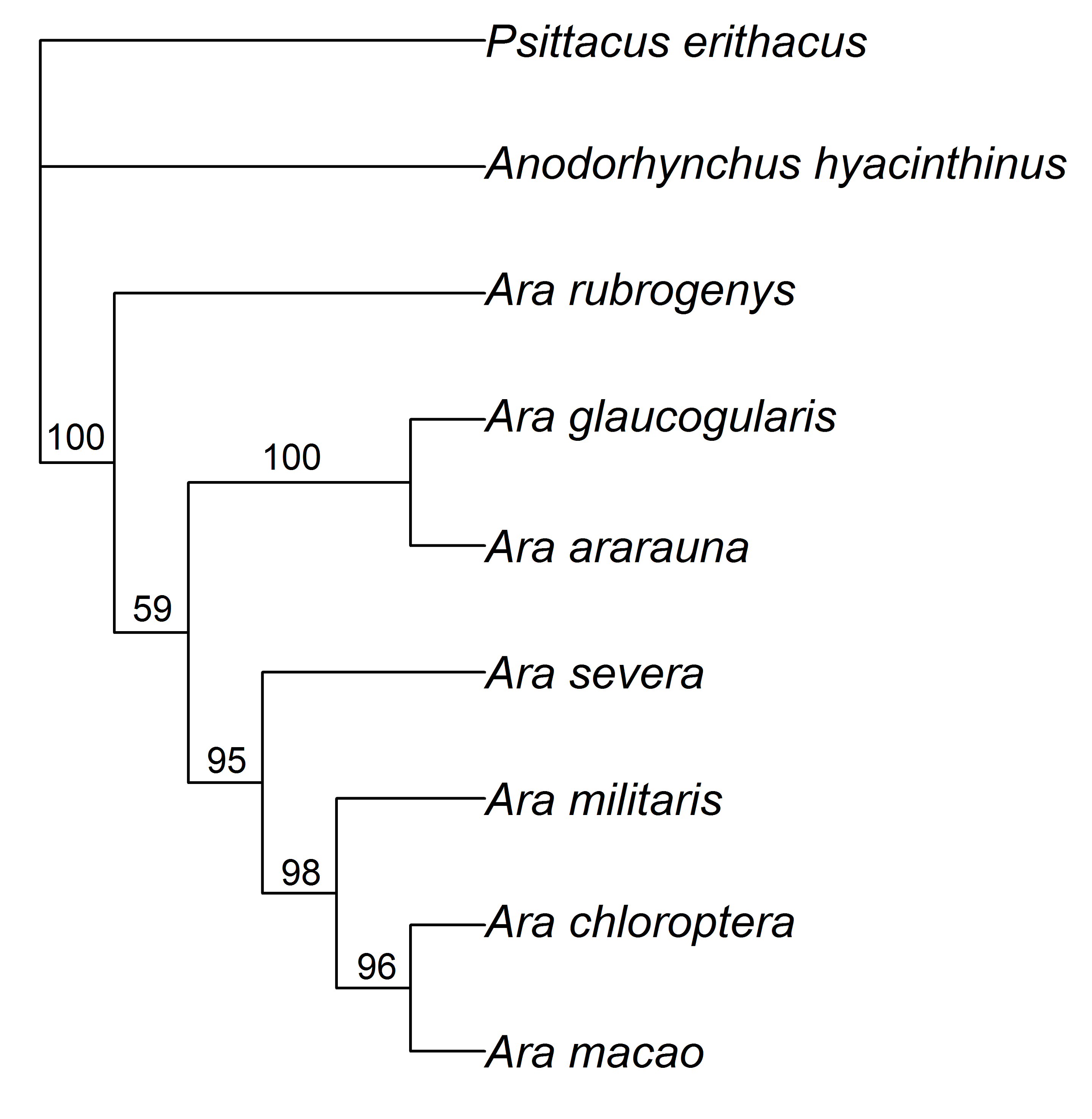

Supplement: Figure S2 — Bayesian tree constructed from a 430 bp RNA 16 s gene fragment for the genus Ara . Posterior probabilities evidence strong support for most branches in the tree. P. erithacus and A. hyacinthinus were used as outgroups. (TIFF) [file pone.0053066.s002.tiff]
